# Food safety and biological control; genomic insights and antimicrobial potential of *Bacillus velezensis* FB2 against agricultural fungal pathogens

**DOI:** 10.1371/journal.pone.0291975

**Published:** 2023-11-14

**Authors:** Masooma Hammad, Hazrat Ali, Noor Hassan, Abdul Tawab, Mahwish Salman, Iqra Jawad, Anne de Jong, Claudia Munoz Moreno, Oscar P. Kuipers, Yusra Feroz, Muhammad Hamid Rashid

**Affiliations:** 1 Industrial Biotechnology Division, National Institute for Biotechnology and Genetic Engineering College, Pakistan Institute of Engineering and Applied Sciences (NIBGE-C, PIEAS), Faisalabad, Pakistan; 2 Health Biotechnology Division, National Institute for Biotechnology and Genetic Engineering College, Pakistan Institute of Engineering and Applied Sciences (NIBGE-C, PIEAS), Faisalabad, Pakistan; 3 Department of Biochemistry, Government College University Faisalabad (GCUF), Faisalabad, Pakistan; 4 Groningen Molecular Biology and Biotechnology Institute (GBB), University of Groningen, Groningen, The Netherlands; Gauhati University, INDIA

## Abstract

Development of natural, broad-spectrum, and eco-friendly bio-fungicides is of high interest in the agriculture and food industries. In this context, *Bacillus* genus has shown great potential for producing a wide range of antimicrobial metabolites against various pathogens. A *Bacillus velezensis* strain FB2 was isolated from an agricultural field of National Institute for Biotechnology and Genetic Engineering (NIBGE) Faisalabad, Pakistan, exhibiting good antifungal properties. The complete genome of this strain was sequenced, and its antifungal potential was assayed by dual culture method. Moreover, structural characterization of its antifungal metabolites, produced *in vitro*, were studied. Genome analysis and mining revealed the secondary metabolite gene clusters, encoding non-ribosomal peptides (NRPs) production (e.g., surfactin, iturin and fengycin) and polyketide (PK) synthesis (e.g., difficidin, bacillaene and macrolactin). Furthermore, the *Bacillus velezensis* FB2 strain was observed to possess *in vitro* antifungal activity; 41.64, 40.38 and 26% growth inhibition against major fungal pathogens i.e. *Alternaria alternata*, *Fusarium oxysporum* and *Fusarium solani* respectively. Its lipopeptide extract obtained by acid precipitation method was also found effective against the above-mentioned fungal pathogens. The ESI-MS/MS analysis indicated various homologs of surfactin and iturin-A, responsible for their antifungal activities. Overall, this study provides a better understanding of *Bacillus velezensis* FB2, as a promising candidate for biocontrol purposes, acting in a safe and sustainable way, to control plant pathogens.

## Introduction

Resistance of pathogenic fungi to antifungal agents in medical and agriculture sectors has been increased globally due to the limited availability of safe and effective antifungal agents [1]. The emergence of antifungal resistant fungi is happening on a much faster track than development of novel antifungal agents, presenting severe human health and food security issues [2]. In agriculture, fungal pathogens are one of the most destructive biotic stresses faced by plants with more than 10,000 fungal species actively involved in causing infection in plants [3]. *Fusarium* and *Alternaria* genus are among the most commonly infesting fungi causing *Fusarium* wilt, *Fusarium* root rot, *Alternaria* early blight, *Alternaria* leaf spot in diverse array of plants [4]. Although a variety of fungicides are present for field applications, such as azoles, prompt concerns of rising antifungal resistance is getting alarming. For example, *Botrytis cinerea* has been reported to be present in strawberry fields across the world [5]. This pathogen is responsible for causing grey mould disease in more than 240 different species of fruits, vegetables and ornamental flowers. It has been showing resistance to multiple fungicides such as benzimidazoles, boscalid, pyraclostrobin, fenhexamid, cyprodinil and fludioxonil [5, 6]. Scientific communities urge to minimize the use of fungicides in agriculture to prevent resistance in pathogenic fungi, but they are still widely used to avoid crop losses, since suitable alternatives are absent. Therefore, the need to develop broad spectrum antifungal agents, possessing nonhazardous nature and safe to human and environment, is in high demand.

Bacteria have great potential to be used as biological control agents and their active metabolites have been proven as a sustainable strategy to control plant diseases and to compete against resistance in pathogenic fungi [7, 8]. Among these bacteria, the genus *Bacillus* is considered a potential source of bioactive antifungal metabolites, used broadly as biocontrol agents against plant pathogens [7]. Species of *Bacillus* have been declared as GRAS (generally recognized as safe) by the US Food and Drug Administration (US FDA) for use in food sectors. For example, *Bacillus subtilis* was reported producing highly active antifungal metabolites particularly effective against multidrug-resistant *Botrytis cinerea* [9], *Bacillus amyloliquefaciens* was observed preventing fruit diseases and plant pathogens e.g., *Botrytis cinerea*, *Monilinia fructicola*, *Penicillium digitatum* and *Penicillium italicum* [9, 10]. Further, growth of *Alternaria alternata*, *Botrytis cinerea*, *Fusarium oxysporum* and *Phytophthora infestans* was inhibited by compounds produced by *Bacillus halotolerans* [11]. In the recent years, *Bacillus velezensis* has been investigated widely for commercial purposes e.g., by using it as biocontrol agent and biofertilizer in agriculture [7, 8, 12].

Therefore, *Bacillus velezensis* is considered a valuable asset producing a variety of compounds such as antifungal bioactive compounds (e.g., lipopeptides) [13], enzymes [14], and volatile compounds [15], and additionally to perform well as plant growth-promoting rhizobacterium (PGPR) [16], hence suppressing fungal pathogenicity and promoting plant growth. *Bacillus velezensis* produced volatile organic compounds, efficiently inhibiting spore germination of *Botrytis cinerea* and mycelial growth of *Alternaria solani* [17]. Earlier, this specie was found to prevent growth and germination of fungal mycelia and spore, respectively, of *Colletotrichum gloeosporioides* by producing cyclic tetrapeptides and lytic enzymes [18]. The synthesis of antifungal metabolites is either ribosomal based such as sulphur antibiotics and antagonistic protein [19] or non-ribosomally based such as lipopeptide antibiotics, and volatile organic compounds [20]. For example, synthesis of lipopeptides possessing antifungal activity was based on nonribosomal peptide synthetases (NRPSs) in *Bacillus velezensis* [21]. Furthermore, the biosynthetic genes responsible for bioactive metabolites production exist in clusters constituting a considerable part of the genome and are involved in diverse biological functions [22]. Previous studies done for understanding the diversity of these gene clusters and for exploring their role in interaction and development of *Bacillus* revealed that the presence of highly conserved gene clusters are related to the functional regulations while less preserved gene clusters are helpful in interaction with other organisms [23, 24].

The present study was aimed to assess the antifungal potential of *Bacillus velezensis* FB2 against various phytopathogens. This study includes complete sequencing of *Bacillus velezensis* FB2 genome and genes mining, exploring secondary bio-active metabolites of *Bacillus velezensis* FB2. Furthermore, antifungal properties and identification of lipopeptides produced by *Bacillus velezensis* FB2 were also included in this study.

## Material and methods

### Selection of FB2 and cultivation conditions

Soil samples were collected from agricultural fields, situated in the National Institute for Biotechnology and Genetic Engineering (NIBGE) Faisalabad. Prior to isolation, 1g soil was heated at 80°C for 20 minutes in order to kill non-spore forming bacteria [25]. Then, dilutions of the treated soil sample were prepared and plated on *Luria-Bertani (LB) agar* medium (g/L: Tryptone 10, Sodium chloride 5, Yeast extract 5, Agar 15) and incubated at 30°C for 48 hours [26]. Morphologically distinct *Bacillus* like colonies were purified by streak plate method on LB agar. Purified colonies were stored at -80°C, as 20% glycerol stocks for subsequent use. Moreover, morphological characteristics of bacterial isolates were examined using standard procedures under a ZEISS Axioscope Plus 2 microscope.

### *In vitro* primary antimicrobial assays

A total of 9 isolates were preliminary screened for their activities against various agricultural and human pathogens. The organisms tested included fungi (*Fusarium culmorum* PV, *Botrytis cinerea* B05.10, and *Verticillium dahlia* JR2), bacteria (*Erwinia carotovora* subsp. *brasiliensis* LMG21371, *Pseudomonas syringae* pv. tomato DC300, *Klebsiella pneumonia*, *Escherichia coli* WA321 and *Bacillus cereus* ATCC14579), oomycete (*Pythium ultimum* P17) and yeast pathogens (*Candida albicans*); all pathogens and the protocol followed has been described by Muñoz CY, et al., (2022) [27]. An overnight grown culture of FB2 isolate (1 × 108 cells/mL) calculated by UV-VIS Spectrophotometer (Dynamica) at OD_600_ = 1 was used for all assays. Briefly, 5 mm^2^ agar plugs of tested fungi were excised from 3–5 days old plates of Potato Dextrose Agar (PDA) (g/L: Dextrose 20, Potato extract 4, Agar 15) and put in the center of fresh PDA plates and 5 μL of all isolates were put at 2 cm distance and incubated at 28 ± 2°C for 3–5 days. For antibacterial assays, bacterial pathogens were grown overnight and mixed with LB agar at the concentration 1 × 10^6^ cells/mL and 5 μL cultures were inoculated and incubated at 33 ± 2°C for 24 hours. Based on the results of primary screening, the FB2 isolate was further processed.

### Complete genome sequencing and assembly

*Bacillus velezensis* FB2 was cultivated in LB broth and incubated at 130 rpm at 30°C for 48 hours. Genomic DNA was extracted using GenElute bacterial genomic DNA kit (Sigma-Aldrich, Munich, Germany) according to the manufacturer’s instructions. Paired end sequencing (PE150) of high quality isolated DNA was done by the Beijing Genomics Institute (BGI) European Genome Center in Denmark on a BGISEQ-500 platform. Whole-genome sequencing libraries were constructed with the MGIEasy universal DNA library prep set (MGI Tech Co., Ltd., Shenzhen, China), which is specifically designed for MGI high-throughput sequencing platform series. From raw reads, low quality reads, contamination and adapter sequences were removed using Trimmomatic version 0.38 and quality of clean reads was examined through FastQC version 0.11.9. To assemble the short reads, Unicycler version 0.4.8 integrated with SPAdes version 3.14.0 was used. Table 1 shows sequence data of FB2 strain and bio-project/bio-sample information is available at NCBI.

**Table 1:** NCBI submission information of FB2 whole genome sequencing data.

Description	Information
Submission ID	SUB11993493
BioProject	PRJNA879316
BioSample	SAMN30804782
Accession no.	JAOCND000000000
Organism	*Bacillus velezensis* FB2

### Phylogenomic comparison and tree

To assess the presence of any contaminating DNA sequence, genome sequence was analyzed by ContEst16S algorithm of EzBiocloud (https://www.ezbiocloud.net/tools/contest16s) [28]. The phylogenetic tree based on the complete genome sequence (https://tygs.dsmz.de/) was constructed using Type (Strain) Genome Server (TYGS) [29]. Genome BLAST Distance Phylogeny (GBDP) distances were calculated from genome sequences and inferred with FastME [30]. GBDP distance formula d5 was used for scaling branch lengths of tree. For the estimation of genomic digital DNA Hybridization (dDDH), Genome-to-Genome Distance Calculator (GGDC) DSMZ (https://www.dsmz.de/services/online-tools/genome-to-genomedistance-calculator-ggdc) was used. For Average Nucleotide Identity (ANI) estimation, ANI calculator was used (https://www.ezbiocloud.net/tools/ani) [31]. For taxonomic placement of an isolate in specie, 70% dDDH and 95–96% ANI is regarded as acceptable threshold [30]. Two tools, NCBI Prokaryotic Genome Annotation Pipeline (PGAP) 6.2 and Rapid Annotation using Subsystem Technology (RAST) server version 2.0 was used for genome annotation [32].

### Prediction of secondary metabolite clusters

The final FASTA file of FB2 was subjected to be analyzed by antiSMASH (antibiotics and Secondary Metabolite Analysis Shell) bacterial version. The webserver antiSMASH was used to predict the genes involved in secondary metabolites synthesis [33]. The antiSMASH webserver works by combining various available genetics data, antimicrobial metabolites and biosynthetic gene clusters to calculate the place and possible function of the gene clusters.

### *In vitro* antagonistic activity of FB2

The tested fungal strains used for the antagonistic activity, were acquired from the First (Fungal) Culture Bank of Pakistan (FCBP), Lahore Pakistan under the accession numbers FCBP-SF-1175, FCBP-PTF-791, FCBP-PTF-1174 for *Fusarium oxysporum*, *Fusarium solani*, *Alternaria alternata* respectively. All the strains were maintained on Potato Dextrose Agar (PDA) at 30 ± 2°C. Dual culture technique was used for screening the antagonistic activity of FB2 strain against fungal pathogens [34]. Briefly, 5 mm fungal plug of each fungal strain was cut from 5–7 days old PDA plates and put in the center of fresh PDA plate. From overnight grown culture of FB2 (OD_600_-1) in LB broth, 20 μL culture was put on both sides of fungal plug at 2 cm distance in test plates. Plates with fungal plug without the inoculation of bacteria served as control. Experiment was done in triplicates. All the plates were kept at 30 ± 2°C for 3 days. Percentage fungal inhibition was measured by the following formula [35]:

$$I\;(\%)=\frac{Mean\;diameter\;of\;fungal\;colony\;in\;control-Mean\;diameter\;of\;fungal\;colony\;in\;test}{Mean\;diameter\;of\;fungal\;colony\;in\;control\;}\times 100$$

### Lipopeptides production and extraction from FB2

For the production of lipopeptides, a single colony of the FB2 strain was inoculated in LB medium and incubated overnight (14–16 hours) at 30°C and 180 rpm) until OD_600 nm_ = 1 was obtained. Next day, the culture was inoculated in 2% in lipopeptides production medium (g/L: glucose 2, monosodium glutamate 1, yeast extract 0.3, MgSO_4_ 0.1, K_2_HPO_4_ 0.1, KCl 0.05, pH 7 ± 0.5) and kept at 30°C and 180 rpm [36]. After 96 hours, culture was centrifuged at 6000 rpm for 15 minutes and cell free supernatant was collected and acidified with 6 M HCl until acquiring pH 2 and kept at 4°C for overnight. Precipitates of lipopeptides were obtained by centrifugation at 7000 rpm for 20 minutes, and dissolved in methanol for further analysis.

### Antifungal activity of lipopeptide extract

The antifungal activity of lipopeptide of methanol extract was assessed using the method described by Zhou L, Song C, Li Z, and Kuipers OP (2021) [37] for bacterial strain with slight modifications. The spores were collected from 5 days old culture of fungal pathogens (*Fusarium oxysporumi*, *Fusarium solani*, *Alternaria alternata*) and washed with sterile water. The spore suspension was prepared at 1 x 10^6^ spores/mL and mixed with PDA which was pre-cooled at 55°C. After mixing, PDA medium was poured in petri dishes and was let to solidify. Two wells of 5 mm^2^ each were made at equal distances from the edge of the plate for each fungal strain with the help of sterile 1 mL tip. 100 μL of methanol extract of lipopeptides was poured in one well and 100 μL methanol was added in the other well as a control. The experiment was done in triplicate. Plates were incubated for 2 to 3 days at 30°C and inhibition zones were measured. The 30°C temperature and 3 days’ incubation time were the optimum growth parameters, which were found best for maximum fungal proliferation to get visible results.

### ESI-MS/MS analysis of lipopeptide extract

In order to find out and characterize the compounds responsible for antifungal activity, lipopeptides methanol extract of FB2 was subjected to Liquid chromatography / mass spectrometry analysis. Mass spectrometric analyses of methanol extract of FB2 strain were done on LTQ XL Linear Ion Trap Mass Spectrometer (Thermo Scientific, USA) equipped with an ESI source. Samples were injected with the help of a syringe pump and the flow rate was 5 μL/min. Source voltage was 4.80 kV and capillary voltage was 23 V in positive ion mode. In both positive and negative scan modes, the capillary temperature and sheath gas (N_2_) flow were 350°C and 30 arbitrary units respectively. The data acquisition was conducted in full scan mode ranging *m/z* 50–2000. Tandem mass spectrometry was further conducted. Both the [M + H]^+^, [M + Na]^+^ in positive mode as well as [M—H]^−^ ions in negative mode were monitored, for the proper characterization of lipopeptides in the ESI-mass spectra and for the confirmation of the structures of lipopeptides, identified peaks were further fragmented by ESI-MS/MS [38].

## Results

### Selection of the FB2 isolate and primary screening

Based on their morphological characters, 9 bacterial isolates (P1, P2, P3, M1, M6, M7, FB1, FB2, and FB3) were selected and subjected to *in vitro* antimicrobial assays. Morphological characteristics included rod-shaped, oblong endospores producing Gram-positive bacterial species, confirmed by ZEISS Axioscope plus 2 microscope. Out of all 9 isolates, the FB2 isolate showed the most promising antimicrobial activity against all tested bacterial and fungal pathogens except *Klebsiella pneumoniae*. Based on the best performance by exhibiting antimicrobial activity, The FB2 isolate was selected for subsequent experiments. Table 2 shows antimicrobial activity of all isolates against tested pathogens.

**Table 2:** Antimicrobial activity screening of bacterial isolates.

Pathogen	Bacillus strains									
	168	P1	P2	P3	M1	M6	M7	FB1	FB2	FB3
*Fusarium culmorum*	-	+	++	++	+	++	++	++	++	-
*Botrytis cinerea*	-	-	-	+	+	-	+	++	++	-
*Verticillium dahliae*	-	+	+	+	+	+	++	+++	+++	+
*Pythium ultimum*	-	++	-	++	+	+	+	++	+++	-
*Erwinia carotovora subsp*. *brasiliensis*	+	+	++	+	+++	+	+++	++	+++	-
*Pseudomonas syringae*	-	-	+++	-	+	+++	++	+++	+++	-
*Klebsiella pneumoniae*	-	-	-	-	-	-	-	-	-	-
*E coli*	-	-	+	-	++	+	+++	+++	+++	-
*Bacillus cereus*	-	-	++	-	-	+	+	++	+++	-
*Candida albicans*	-	-	-	-	-	-	++	++	++	-

(-) No activity, (+) low activity, (++) moderate activity, (+++) high activity

### Genome assembly and annotation

A total of 4.6 M paired-end clean reads (100 bp) were obtained from genome sequencing. The *de novo* assembly of the genome was comprised of 13 contigs and genome coverage was 200 X. The genome annotation of *Bacillus velezensis* FB2, based on Classic RAST, revealed that the genome was comprised of 3,888,040 bp (3.8 Mb) and possessed other key characteristics including 46.4% GC content, N50 (2058779) and L50 (1). The total 13 contigs were counted in the genome sequence of FB2 isolate and it was comprised of 462 subsystems, 3965 protein-coding sequences, and 58 RNAs and 52 tRNAs. Table 3 shows general genomic features possessed by *Bacillus velezensis* FB2.

**Table 3:** Genomic attributes of FB2 strain.

Features	Values
Genome size	3,888,040
GC Content	46.4
N50	2058779
L50	1
Number of Contigs	13
Number of Subsystems	462
Number of Coding Sequences	3965
Number of RNAs	58
tRNA	52

**Fig 1:**
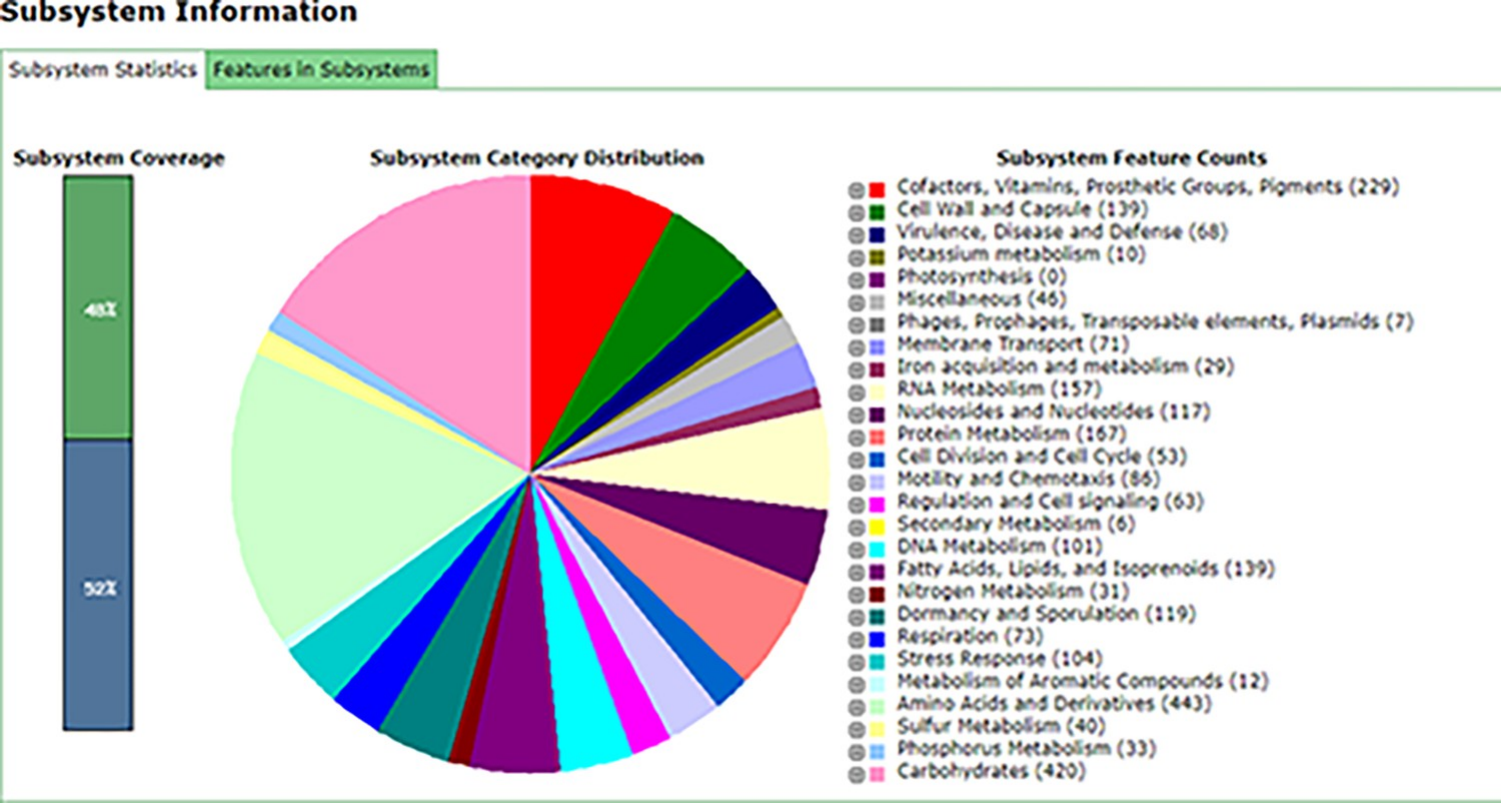
Subsystem information retrieved from Classic RAST indicating different subsystems found in the genome of FB2.

Further, based on the functional characteristics, 462 subsystems present in the genome represented 229 ORFs responsible for cofactors, vitamins, prosthetic groups, pigments. While, 139 ORFs for cell wall and capsule, 68 ORFs for virulence, disease and defense, 157 ORFs for RNA metabolism and various others are included in the genome of FB2 strain. Fig 1 shows subsystems information of FB2 obtained from Classic RAST.

### FB2 identification and phylogenomic analysis

Table 4 shows the dDDH and ANI values for individual bacteria, obtained from TYGS and ANI calculator, respectively.

**Table 4:** Percentage dDDH and ANI of FB2 with other related strains.

Strain name	dDDH (%)	ANI (%)
*Bacillus methylotrophicus* KACC 13105	99.9	98.23
*Bacillus velezensis* NRRL B-41580	84.4	98.23
*Bacillus siamensis* KCTC 13613	56.5	94.36
*Bacillus nakamurai* NRRL B-41091	30.7	86.35
*Bacillus inaquosorum* KCTC 13429	20.8	77.5
*Bacillus vallismortis* DV1-F-3	20.2	76.85

**Fig 2:**
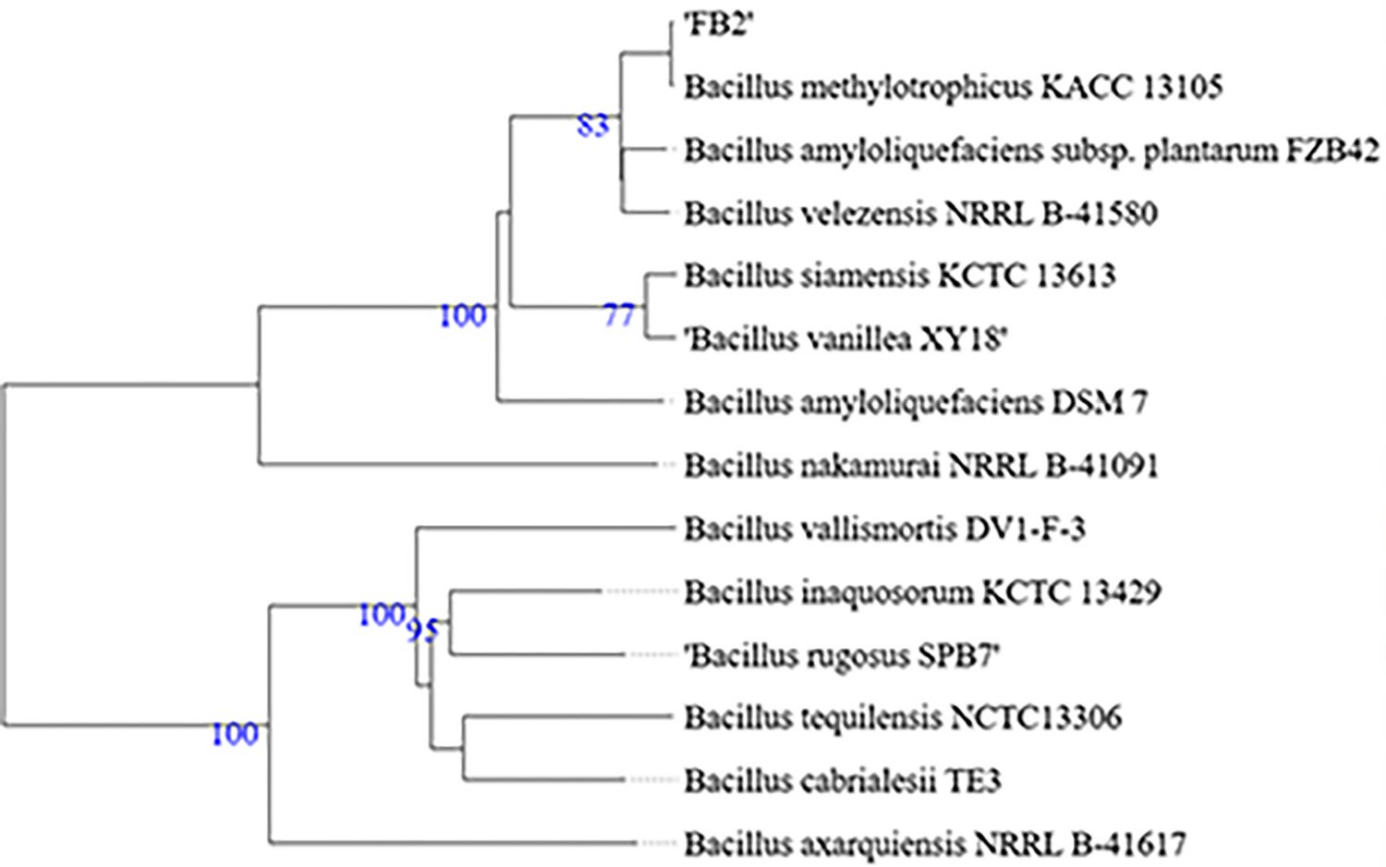
Phylogenetic tree for FB2 based on whole genome generated by type (Strain) genome server. Pseudo bootstrap values were set > 60% from 100 replications in Genome BLAST Distance Phylogeny (GBDP).

After comparing with multiple strains including 4 major species of *Bacillus subtilis* group, TYGS analysis showed 99.9% dDDH and ANI calculator gave 98.23% similarity of FB2 strain to *Bacillus methylotrophicus* KACC 13105 (which is synonymous to *Bacillus velezensis* [39]). Further, by NCBI BLAST, 16S gene of FB2 was found highly similar to *Bacillus velezensis* strain FZB42. Fig 2 shows the phylogenetic tree, based on the whole genome sequence, by GBDP showed relatedness of the bacteria.

### Prediction of genes responsible for secondary metabolism

Through antiSMASH analysis, 12 regions were detected in the genome of the FB2 strain. Out of which, 3 belonged to NRPS i.e. (Fengycin, surfactin and bacillibactin), 2 to Trans-PK clusters (macrolactin H and difficidin,), 1 to TypeIII PKS (unknown) 1 to PK like (butirosin), 1 hybrid NRPS/PKS (bacillaene), 2 terpenes (unknown), 1 belonged to lanthipeptide-class-ii like cluster and one was uncharacterized (similar to bacilysin. Table 5 shows the summary of biosynthetic gene clusters detected by antiSMASH analysis. A large DNA sequence of 37 kb is devoted for biosynthesis of surfactin in the FB2 strain. There were three core genes, *srf AA*. *srfAB*, *srf AC* and an external thioesterase named *srf D* found, which had 99–100% identities to the same genes found in *Bacillus amyloliquifaciens* or *Bacillus velezensis*. The operon for iturin synthesis (37 kb) was found to be highly similar to that of *Bacillus spp*. (95–98%), consists of 4 core genes. *ituD* encodes a malonyl-coA transcyclase mediating the incorporation of fatty acid chain in iturin structure, while other three genes *ituA*, *ituB*, *ituC* are involved in amino acid integration. Further, a five genes containing cluster (*fenA-E*) (28 kb) responsible for fengycin production, was identified which was 95–99% similar to other *Bacillus* spp. Regarding polyketide synthetases, three gene clusters were identified; for the biosynthesis of antibacterial compounds difficidin, bacillaene and macrolactin with amino acid similarities between 95–99% to other *Bacillus* spp.

**Table 5:** Biosynthetic gene clusters with predicted products, found in the genome of FB2, by antiSMASH analysis.

Region	Size (nt)	Cluster Type	Compund
Region 1	41,245	PKS-like	butirosin A / butirosin B
Region 2	17,409	terpene	Unknown
Region 3	28,889	lanthipeptide-class-ii	Unknown
Region 4	87,836	transAT-PKS	macrolactin H
Region 5	100,566	transAT-PKS,Type III PKS,NRPS	bacillaene
Region 6	137,722	NRPS,transAT-PKS,betalactone	fengycin
Region 7	21,884	terpene	Unknown
Region 8	41,101	Type IIIPKS	
Region 9	93,793	transAT-PKS	difficidin
Region 10	41,419	other	bacilysin
Region 11	51,792	RiPP-like,NRPS	bacillibactin
Region 12	65,408	NRPS	surfactin

### *In vitro* antagonistic activity of FB2 strain

**Fig 3:**
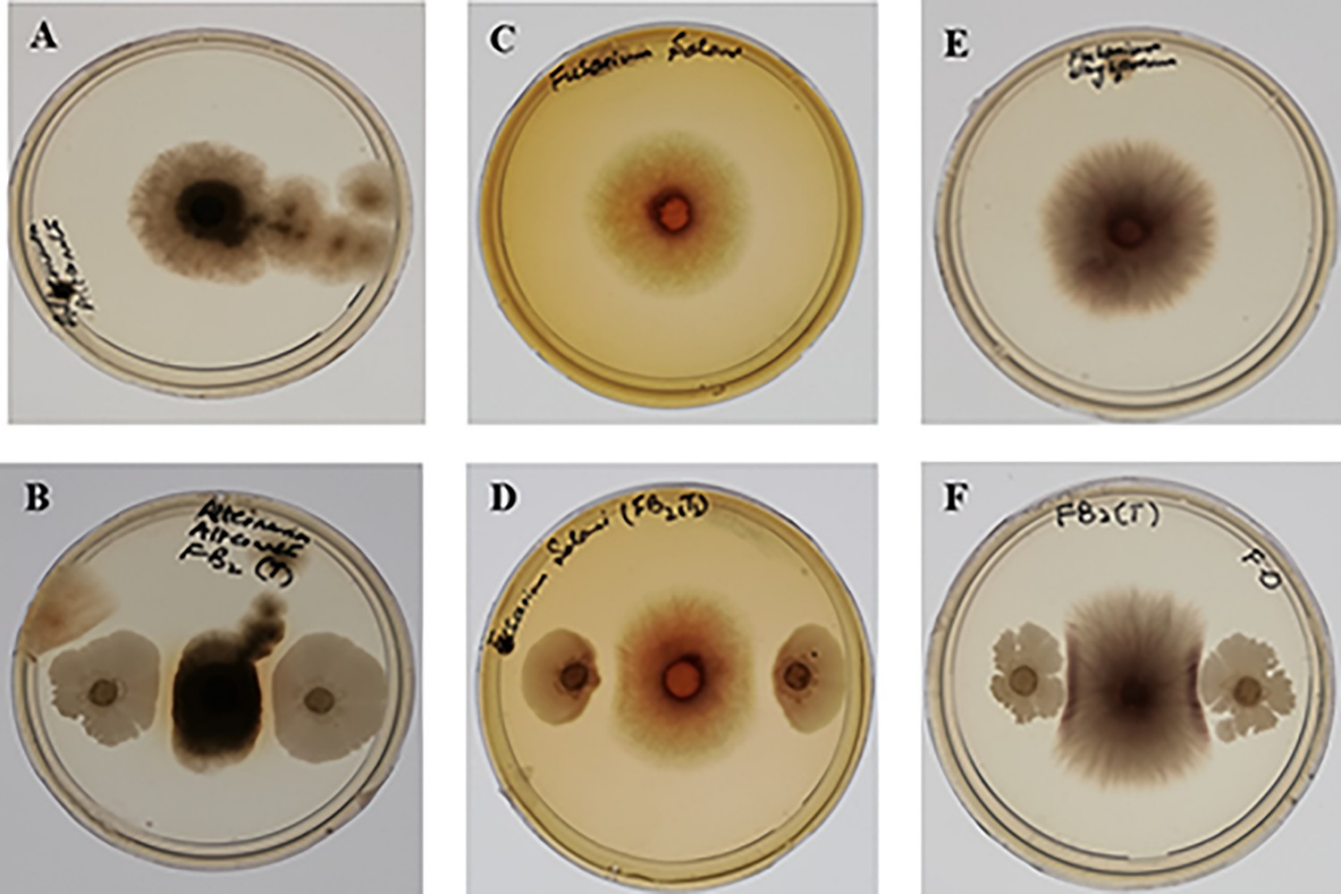
Antagonistic activity of FB2 strain against fungal pathogens. **A.** Control of *Alternaria alternata* B. Test of *Alternaria alternata*. **C**. Control of *Fusarium solani* **D**. Test of. *Fusarium solani* **E**. Control of *Fusarium oxysporum* **F**. Test of *Fusarium oxysporum*.

The FB2 strain was found to have antagonistic activity against tested plant fungal pathogens confirmed by observing reduced diameters of fungal colonies in test plates as compared to control after 3 days of incubation as shown in Fig 3.

Table 6 shows the growth inhibition of fungal pathogens caused by FB2 strain. Percentage inhibition was found to be highest for *Alternaria alternata* (41.64%) following *Fusarium oxysporum* (40.38%), then *Fusarium solani* (26%).

**Table 6:** Percentage inhibition of fungal pathogens by FB2 strain.

Fungal pathogen	Colony diameter in control (cm)	Colony diameter in test (cm)	Inhibition (%)
*Alternaria alternata*	3.53 ± 0.14	2.07 ± 0.21	41.64
*Fusarium oxysporum*	4.63 ± 0.08	2.77 ± 0.03	40.38
*Fusarium solani*	4.10 ± 0.10	3.03 ± 0.08	26

Presented values are the means± standard error of mean

### *In vitro* antifungal activity of lipopeptides extract

**Fig 4:**
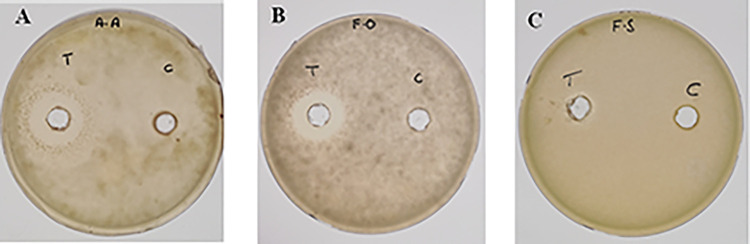
Antifungal assays of methanol extracts of FB2 against fungal pathogens. C = Methanol as control, T = Lipopeptides extract of FB2, **A**. *Alternaria alternata* **B**. *Fusarium oxysporum* **C**. *Fusarium solani*.

The lipopeptides methanolic extract of FB2 strain was found to be active against all the three fungal pathogens; *Alternaria alternata*, *Fusarium oxysporum* and *Fusarium solani* as shown in Fig 4.

Table 7 shows zone of inhibition of test fungal pathogens caused by lipopeptides extract of FB2. Inhibition zone was largest for *Alternaria alternata*, followed by *Fusarium oxysporum* and *Fusarium solani* in decreasing order. This showed that antifungal activity of the FB2 strain was mainly caused by its lipopeptides.

**Table 7:** Zone of inhibition of methanolic extracts against fungal pathogens.

Fungal strain	Zone of inhibition (mm)
*Alternaria alternata*	11 ± 0.5
*Fusarium oxysporum*	7 ± 0.5
*Fusarium solani*	2.7 ± 0.29

Presented values are the means± standard error of mean

### Metabolites analysis by ESI-MS/MS

**Fig 5:**
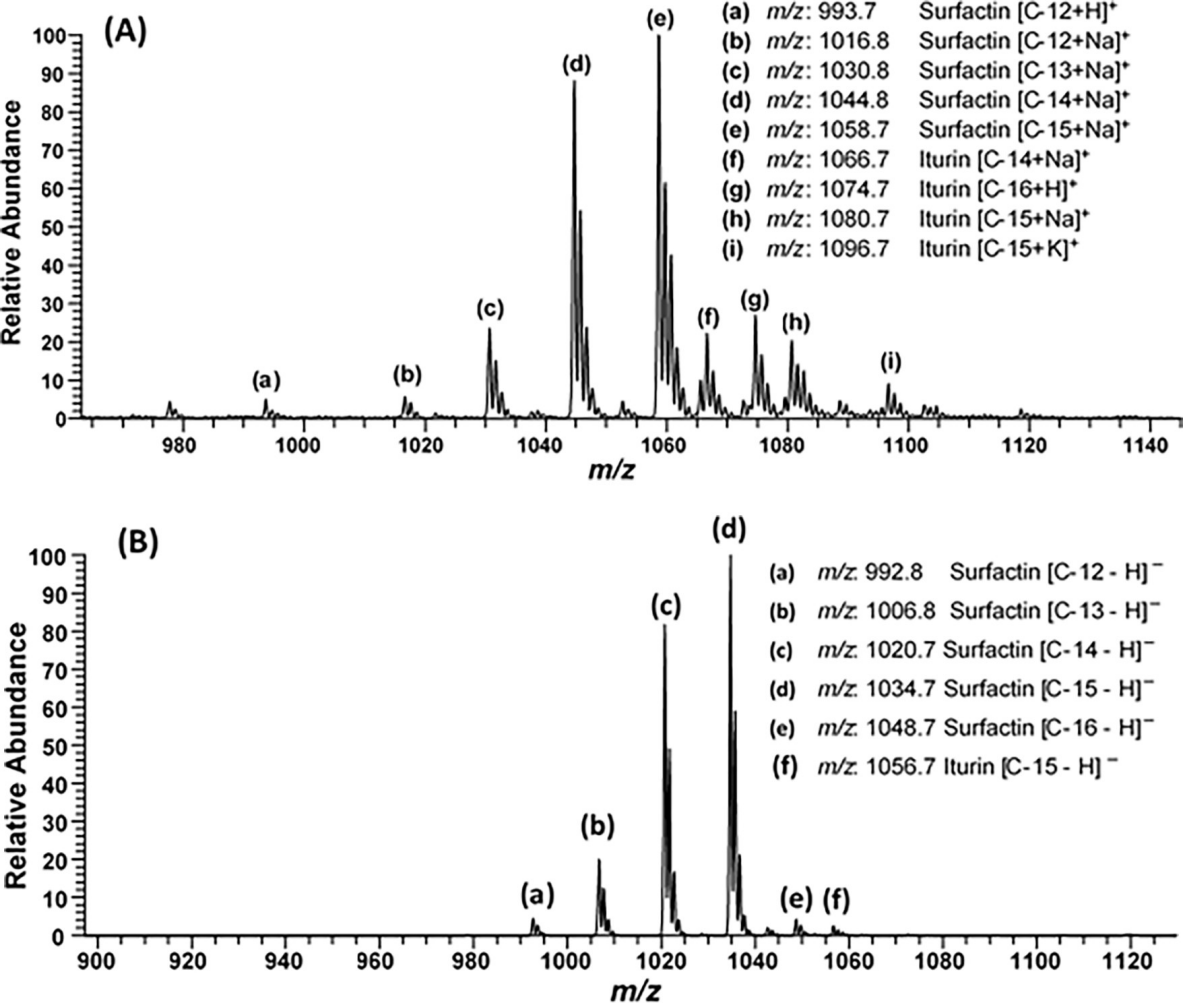
ESI-MS/MS full scan of the metabolites, obtained from FB2, in **(A)** positive ionization mode and **(B)** negative ionization mode.

During the full scan mass spectrometry of methanolic extracts of FB2, two families of products were identified both in positive as well as negative modes. Fig 5A shows detected peaks in positive mode. In positive mode, the observed groups were in the range of *m/z* 993–1058 and *m/z* 1066–1096. All the observed full scan peaks were further subjected to tandem mass spectrometry for further confirmation. The MS^n^ of ion peaks at *m/z* 1016.8, 1030.8, 1044.8 and 1058.7 revealed the sodiated ions of surfactin C-12, C-13, C-14 and C-15, respectively. The ion peaks, at *m/z* 1066.7 and 1080.7 corresponded to sodiated ions of C-14 and C-15 of iturin-A, while the ion peak at *m/z* 1074.7 and 1096.7 were identified as protonated and potassium ion of iturin-A C-16 and C-15, respectively.

Among all the molecular moieties, the sodiated ion peak of surfactin homolog C-15 at *m/z* 1058.7 was found to be the most abundant. The relative abundances of other homologs of surfactin, at ion peaks of *m/z* 993.7, 1016.8, 1030.8, and 1044.8 were 5, 6, 24 and 90% each, for [C12 + H]^+^, [C12 + Na]^+^, [C13 + Na]^+^ and [C14 + Na]^+^ respectively. Relative abundance of different homologs of iturin at ion peaks of *m/z* 1066.7, 1074.7, 1080.7 and 1096.7 was 24, 28, 22 and 10% each, for [C14 + Na]^+^, [C16 + H]^+^, [C15 + Na]^+^ and [C15 + K]^+^ respectively.

In negative mode, also two groups were detected as shown in Fig 5B. One group was in the range of *m/z* 992–1048 and the other was observed on *m/z* 1056.67. Here four homologs of surfactin C-12, C-13, C-14, C-15 and C-16 were detected at *m/z* 992.67, 1006.75, 1020.67, 1034.67 and 1048.67, respectively, while one homolog C-15 of iturin-A at *m/z* 1056.67 was also detected during the MS^2^ of corresponding ion peaks.

In negative mode, the most abundant molecular moiety was C-15 homolog of surfactin, similar to the positive mode. While comparing abundances of other molecules, C-12, C-13, C-14 and C-16 homologs of surfactin were detected at ion peaks of *m/z* 992.8, 1006.8, 1020.7, 1034.7 and 1048.7 and their relative abundances of each were 6, 21, 82 and 6% respectively. The only homolog of iturin found was C-15 at *m/z* 1056.7 which was present 4% as compared to the most abundant molecule.

Table 8 summarizes the MS^n^ data of the detected peaks indicating that they belong to surfactin and iturin classes of lipopeptides [38].

**Table 8:** The ESI-MS/MS of lipopeptides, produced by *Bacillus velezensis* FB2 strain.

Metabolites	Exact mass	Observed peaks (*m/z*)				Homologs
		[M + H]^+^	[M + Na]^+^	[M + K]^+^	[M—H]^-^	
Surfactin	993	993.7	1016.8		992.8	C-12
	1007		1030.8		1006.8	C-13
	1021		1044.8		1020.7	C-14
	1035		1058.7		1034.7	C-15
	1049				1048.7	C-16
	1063					C-17
Iturin	1043		1066.7			C-14
	1057		1080.7	1096.7	1056.7	C-15
	1073	1074.7				C-16

## Discussion

Based on the resilient nature and diverse habitat, the *Bacillus* genus is capable of producing diverse range of bioactive molecules, making this genus an ideal candidate to be used as biocontrol agent. Importantly, species of *Bacillus* and their antimicrobial metabolites are considered safe for humans and the environment. Hence, this study was focused to explore *Bacillus* species as a new biocontrol agent and genomic insights into antifungal potentials of *Bacillus* species. In the present study, a total of 9 bacteria were isolated showing antagonistic activities against different human and agricultural pathogens. Based on showing best antifungal potential, the FB2 isolate was subjected to complete genome sequence for elucidating the underlying genomic information related to broad spectrum functionality and to subsequent *in vitro* experiments, The FB2 isolate was identified as *Bacillus velezensis* based on dDDH and ANI after complete genome sequence.

In the current study, the complete genome sequence analysis of FB2 strain revealed that a considerable amount of the genome was devoted for the production of secondary metabolites possessing broad spectrum of antibacterial and antifungal properties. Previous studies also presented high genomic similarity for *Bacillus velezensis* reported in this study, suggesting this species as ideal candidate for biocontrol [40, 41]. Further, 13 gene clusters were identified in the studied genome, out of which 4 were found to be for synthesis of NRPS (surfactin fengycin, iturin and bacillibactin) and 3 gene clusters belonged to the class of antimicrobial polyketide synthetases (bacillaene, difficidin and macrolactin). These genes clusters have been reported in almost every genome of *Bacillus velezensis* [42] and are responsible for regulating broad antifungal and other antimicrobial activities in *Bacillus velezensis* as well as involved in the suppression of plant pathogens [43]. Moreover, a big cluster (51 kb) for the biosynthesis of bacillibactin has been detected in the genome of *Bacillus velezensis* FB2, suggesting this presence might be attributed to antibacterial activity against agricultural pathogen e.g., *Pseudomonas syringae* [44]. Bacillibactin is siderophore known for scavenging iron from the environment and has been found to have antibacterial potential against plant pathogens including *Pseudomonas syringae* [44]. Further, polyketide synthases (PKS) clusters have been found exhibiting various functions related to plant immunity and antagonism against bacterial plant pathogens and even in inhibition of cancer proliferation [45].

This study also evaluated the antagonistic activities of *Bacillus velezensis* FB2 based on cell-free supernatants and lipopeptide extracts against different phytopathogens. Among the variety of bioactive molecules produced by *Bacillus velezensis*, lipopeptides are known for their excellent antimicrobial performance [46]. The lipopeptide extract was found most effective against *Alternaria alternata*, then *Fusarium oxysporum* and against *Fusarium solani*. Here, we conclude that the antifungal activity of *Bacillus velezensis* FB2 to these fungi can be attributed to lipopeptides production. Previous studies have also explored root colonization capacity and cell motility of lipopeptides once subjected to the extracellular matrix [24, 47]. Further, these molecules have been involved in decreasing superficial tension and inducing systemic resistance in plant [48]. Various *Bacillus velezensis* strains have been exploited for their antagonistic activities against different *Alternaria* and *Fusarium* pathogens *in vitro* and *in vivo* studies [49–52]. Hence, presence of gene clusters revealed by complete genome sequence and *in vitro* demonstration of antifungal activity by *Bacillus velezensis* FB2 showed that lipopeptides are central to making the best choice for being used as biocontrol agents in plants. Although, *in vitro* studies provide valuable preliminary data, it is important to acknowledge the limitations of extrapolating these findings to real-world agricultural settings. Further research and *in vivo* studies are needed to assess the efficacy and practical applicability of *Bacillus velezensis* FB2 as biocontrol agent under field conditions in future.

Mass spectrometric analysis of lipopeptide extracts showed the presence of surfactins and iturins, belong to the class of cyclic lipopeptides (CLP), comprised of α-amino acids linked to fatty acid chain through β-hydroxyl (surfactins or fengycins) or β-amino(iturins) linkage forming amphipathic compounds. Surfactin is a well-known biosurfactant and has been established as promising agent with virtuous antifungal, antiviral, antitumoral and anti-mycoplasma activities [53]. Further, iturin has been found as the most efficient antifungal lipopeptide discovered so far [54]. Fengycin has great potential of specifically inhibiting the growth of filamentous fungi [55]. However, no homolog of fengycin was detected in the culture medium in the present study, which might be due to lower indictable production. By combining the results of antifungal activity shown by crude extract, and ESI-MS analysis showing the presence of surfactin and iturin, this could be inferred that either both of these compounds have their own activities or they may be acting synergistically to inhibit tested fungal growth. The latter observation is in agreement with the previous studies which proposed that surfactin does not have antimicrobial activity by its own, but rather acts synergistically with iturin or other metabolites for enhance antimicrobial activity [56, 57]. In another study, a crude extract of *Bacillus velezensis* DTU001 has been found more effective as compared to individual antifungal compounds (iturins and fengycins) [2]. The individual activities of all compounds remain to be assessed in a future study, as well as the identification of aberrant amino acid residues in the known antifungal compounds, which could give them altered properties.

## Conclusion

*Bacillus velezensis* FB2 showed antifungal activities against various phytopathogens. The surfactins and iturins were the main components of lipopeptides extracted from this strain as confirmed by LC/MS analysis. Complete genome sequence revealed multiple gene clusters responsible for secondary metabolites synthesis, possessing broad spectrum of antibacterial and antifungal properties. The presence of all these significant gene clusters in the genome suggests that this strain has great potential for use in agriculture sector as biocontrol agent.
